# Sex differences in the clinical manifestations related to dependence behaviors in medication-overuse headache

**DOI:** 10.1186/s10194-023-01685-z

**Published:** 2023-11-01

**Authors:** Yen-Feng Wang, Yi-Shiang Tzeng, Chia-Chun Yu, Yu-Hsiang Ling, Shih-Pin Chen, Kuan-Lin Lai, Wei-Ta Chen, Shuu-Jiun Wang

**Affiliations:** 1 Department of Neurology, Neurological Institute, Taipei Veterans General Hospital, Bei-Tou District, No. 201, Sec. 2, Shi-Pai Road, Taipei, 11217 Taiwan; 2 College of Medicine, National Yang Ming Chiao Tung University, Taipei, Taiwan; 3 Brain Research Center, National Yang Ming Chiao Tung University, Taipei, Taiwan; 4 Division of Translational Research, Department of Medical Research, Taipei Veterans General Hospital, Taipei, Taiwan; 5 Department of Neurology, Ministry of Health and Welfare Keelung Hospital, Keelung, Taiwan

**Keywords:** Chronic migraine, Medication-overuse headache, Dependence, Sex differences, Diagnosis, Smoking

## Abstract

**Objective:**

The present study aimed to compare sex differences in the clinical manifestations related to dependence behaviors in medication-overuse headache (MOH).

**Methods:**

Consecutive patients with newly diagnosed chronic migraine (CM) with and without MOH based on the Third Edition of International Classification of Headache Disorders (ICHD-3) were enrolled prospectively from the headache clinic of a tertiary medical center. Demographics and clinical profiles were collected by using a questionnaire, which included current use of tobacco, alcohol, and caffeinated beverages, the Leeds Dependence Questionnaire (LDQ), the Severity of Dependence Scale (SDS), the Headache Impact Test-6 (HIT-6), and the Pittsburgh Sleep Quality Index (PSQI).

**Results:**

In total, 1419 CM patients (1135F/284 M, mean age 41.7 ± 13.9 years) were recruited, including 799 with MOH (640F/159 M, mean age 42.5 ± 13.2 years) (56.3%). Smoking was associated with an increased risk for MOH in men (odds ratio [OR] = 3.60 [95% confidence interval = 1.73–7.50], *p* = 0.001), but not in women (OR = 1.34 [0.88–2.04], *p* = 0.171) (*p* = 0.021 for interaction). Hypnotic use ≥ 3 days/week was a risk factor for MOH (OR = 2.55 [95% confidence interval = 2.00–3.24], *p* < 0.001), regardless of sex. By using receiver operating characteristics (ROC) curves, the cutoff scores of the LDQ for MOH were determined at 7 for women and 6 for men, and those for the SDS were 5 and 4, respectively (area under curve all ≥ 0.83). Among patients with MOH, the male sex was associated with a shorter latency between migraine onset and CM onset (12.9 ± 11.1 vs. 15.4 ± 11.5 years, *p* = 0.008), despite less average headache intensity (6.7 ± 1.9 vs. 7.2 ± 1.9, *p* = 0.005), functional impacts (HIT-6: 63.4 ± 8.3 vs. 65.1 ± 8.0, *p* = 0.009), and sleep disturbances (PSQI: 10.9 ± 4.4 vs. 12.2 ± 4.3, *p* = 0.001).

**Conclusions:**

The current study identified an association between smoking and MOH in men, as well as sex-specific cutoffs of the LDQ and the SDS, for MOH. MOH was characterized by a shorter latency between migraine onset and CM onset in men and a more severe phenotype in women. Sex should be considered as an important factor in the evaluation of MOH.

## Background

Medication-overuse headache (MOH) is one of the leading causes of disease-related disability among all neurological disorders [1]. The estimated prevalence of MOH is about 1% in the general population [2–5], and it is associated with tremendous socioeconomic impacts [1, 5]. Timely diagnosis and management are indispensable in reducing the disease burden of MOH. In particular, it was found in the Chronic Migraine (CM) Epidemiology and Outcomes (CaMEO) study that medication overuse (MO) remained an important issue in about three fourths of CM patients, despite having had medical consultation, accurate diagnosis, and minimally appropriate medical treatment [6]. Therefore, MOH deserves more attention from the general public and medical professionals.

There is a growing interest in sex-related differences in medicine, especially in epidemiology, pathophysiology, clinical manifestations, and treatment response [7]. In clinical neurology and psychiatry, such a topic is gaining attention in headache disorders and substance use disorders (SUDs) [8–10]. Similar to CM, MOH is more common in women than in men [11–13]. However, there is little information about sex differences in the clinical manifestations of MOH. In particular, behaviors of substance dependence were reported to be present in up to two thirds of MOH patients [14–17]. In addition to acute medications, use of tobacco and other psychoactive substances is more common in patients with MOH than in those without [18, 19]. These findings are suggestive of shared pathophysiology with SUDs. Recently, there were reports pointing out the roles of sex-specific mechanisms in reward and addiction [20]. Whether the associations between MOH and clinical manifestations related to dependence behaviors could also be different in women and men is yet to be clarified. Such sex differences could have important clinical implications, and need to be elucidated.

The Leeds Dependence Questionnaire (LDQ) and the Severity of Dependence Scale (SDS) are two neuropsychological instruments commonly used in the clinical studies of dependence behaviors in SUDs [21, 22] and in MOH [23–26]. It was demonstrated that the LDQ and the SDS were useful to detect the presence of medication overuse (MO) or MOH [23–26], and the scores were correlated with the outcomes [17, 27, 28]. However, in many of the studies, comparisons were made between chronic daily headache (CDH) or CM patients with MOH and those with episodic migraine (EM), episodic cluster headache, or episodic tension-type headache (TTH) or even healthy controls. Besides, some of data were from population-based studies, namely the Akershus study [25, 26, 28]. The comparative performance of these two instruments in the detection of MOH among CM patients in the clinical settings is uncertain. More importantly, whether there could be sex differences in the diagnostic utilities of these two instruments for the detection of MOH among CM patients remains to be determined.

The aims of the present study were (1) to determine sex differences in the association between MOH and the current use of tobacco, alcohol, caffeine, or hypnotics, (2) to compare the diagnostic utilities of the LDQ and the SDS in the detection of MOH between women and men with CM, and (3) to evaluate between-sex differences in the clinical presentations of MOH.

## Methods

### Patients

In this prospective study, patients with newly diagnosed CM with and without a concomitant diagnosis of MOH between September 2018 and November 2022 were enrolled consecutively. Patients were recruited at their first visit to the Headache Clinic of Taipei Veterans General Hospital, a tertiary medical center in the capital city of Taiwan. The Taiwan National Health Insurance (NHI) covers > 99% of the population of our country. Although a referral mechanism is included in the Taiwan NHI, it is not mandatory. Most of the medical expenses are reimbursed, and copayment associated with direction consultations with specialists, even in tertiary medical centers, without referral is typically minimal [29].

Headache diagnoses were made by headache specialists according to the diagnostic criteria of the Third Edition of the International Classification of Headache Disorders (ICHD) (ICHD-3) [30]. The inclusion criteria were (a) willingness to participate in the study, (b) age between 20 and 65 years, and (c) fulfillment of the ICHD-3 criteria for migraine. The exclusion criteria included (a) episodic migraine, (b) coexistence of an acute headache disorder (within one month of headache onset), (c) coexistence of a secondary headache disorder, and (d) difficulties completing the history taking or the questionnaire-based interview. The study protocols were approved by the Institutional Review Board of Taipei Veterans General Hospital (TVGH IRB No. 2018–07-020BC, and 2019–07-002CC). All of the patients gave written informed consent before entering the study.

### Questionnaire-based interviews

Demographics, clinical profiles, headache characteristics, and behaviors of dependence were collected by using a specifically designed questionnaire. The current use of tobacco, alcohol, and caffeinated beverages (coffee, tea, Coke, etc.) was queried specifically, and the responses were treated as binary variables. The 6-item Headache Impact Test (HIT-6) was used to measure the negative impact on daily activities resulting from headache [31]. The Hospital Anxiety and Depression Scale (HADS) was used to screen for psychological disturbances, and included subscales for anxiety (HADS-A) and depression (HADS-D) [32]. The quality of sleep was assessed by using the Pittsburg Sleep Quality Index (PSQI) [33], which included a question for the status of hypnotic use (none, < 1 day/week, 1–2 days/week, ≥ 3 days/week). Frequent hypnotic use was defined as hypnotic use ≥ 3 days/week, the frequency of which was close to the definition of MOH on the ICHD-3 [30]. The severity of dependence behaviors was rated by using modified versions of the LDQ and the SDS for the use in headache disorders [21–23, 25]. The scores of these instruments were verified by headache specialists at face-to-face interviews.

The modified version of LDQ consists of ten questions [23], each of which is to be rated on a scale of 0 to 3 (0 = never, 1 = sometimes, 2 = often, 3 = nearly always). The total score ranges from 0 to 30. The questions are as follows: 1. Do you find yourself thinking about when you will next be able to take analgesics? 2. Is taking analgesics more important than anything else you might do during the day? 3. Do you feel your need for analgesics is too strong to control? 4. Do you plan your days around taking analgesics? 5. Do you take analgesic in a particular way in order to increase the effect it gives you? 6. Do you take analgesics morning, afternoon and evening? 7. Do you feel you have to carry on taking analgesics once you have started? 8. Is getting the effect you want more important than the particular analgesic you use? 9. Do you want to take more analgesics when the effect starts to wear off? 10. Do you find it difficult to cope with life without analgesics?

There are five questions in the modified version of SDS [25], and each item is graded and scored between 0 and 3. The score “0” denotes never/almost never for questions 1–4 and not difficult for question 5, and the score “3” indicates always/nearly always for questions 1–4 and impossible for question 5. The range of the total score lies between 0 and 15. The questions are as follows: 1. Do you think your use of your headache medication is out of control? 2. Does the prospect of missing a dose make you anxious or worried? 3. Do you worry about your use of your headache medication(s)? 4. Do you wish you could stop? 5. How difficult do you find it to stop or go without your headache medication?

### Statistical analysis

Continuous variables were compared by using the Student’s t test between groups, or the Mann–Whitney U test if the null hypothesis of normality was rejected. Categorical variables were compared by using the chi-square test. The optimum cutoff scores of the LDS and SDS for detecting the presence of MOH were determined by using receiver operating characteristics (ROC) curves, in conjunction with Youden’s J statistics. The discriminative performances of these two instruments were evaluated by the areas under the ROC curves (AUC). In general, an AUC of 0.7 to 0.8 indicates acceptable, 0.8 to 0.9 excellent, and > 0.9 outstanding accuracy of a diagnostic test [34]. Logistic regression modeling (enter method) was carried out to estimate the odds ratios (ORs) and the 95% confidence intervals (CIs) for having MOH in relation to the status of smoking, before and after taking potential confounders into consideration. Statistical analysis was carried out by using IBM SPSS Statistics for Windows, version 24.0 (IBM Corp., Armonk, NY, USA). Statistical significance was defined as a two-sided *p* of < 0.05.

## Results

### Study participants

**Fig. 1:**
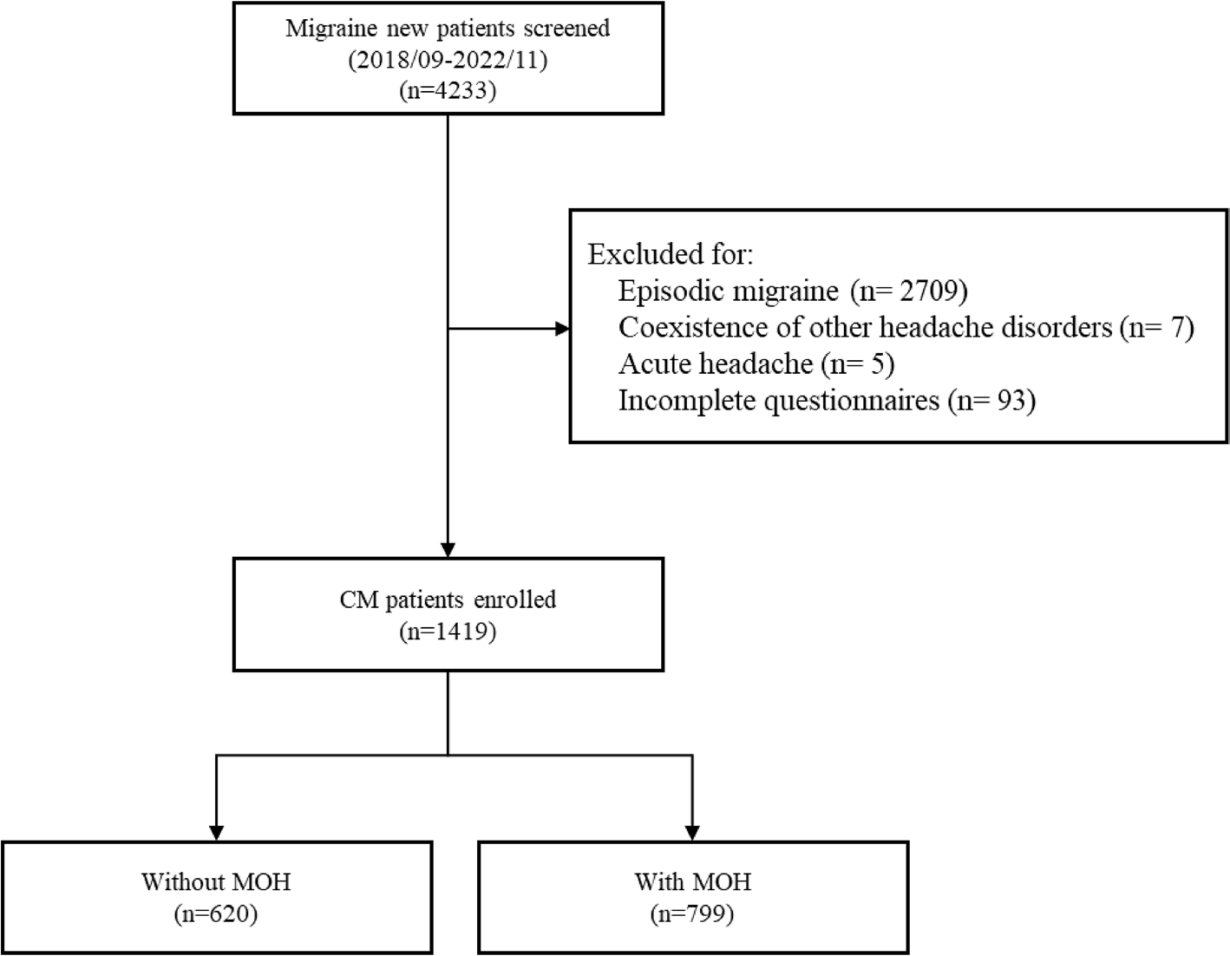
Flow chart of patient recruitment

During the study period, 4233 consecutive migraine patients were screened at their first visit, and 2814 patients were excluded for being diagnosed as episodic migraine (*n* = 2709), coexistence of other headache disorders (*n* = 7) or acute headache (*n* = 5), or incomplete questionnaires (*n* = 93) (Fig. 1). In total, 1419 CM patients (1135F/284 M, mean age 41.7 ± 13.9 years) were included in the analysis, 799 of which had a coexisting diagnosis of MOH (56.3%) (640F/159 M, mean age 42.5 ± 13.2 years) (Table 1). Among patients with MOH (*n* = 799), acute medications being overused were multiple medications in 439 (54.9%), acetaminophen in 167 (20.9%), combination analgesics in 81 (10.1%), nonsteroidals in 25 (3.1%), cold syrups in 19 (2.4%), triptans in 13 (1.6%), tramadol in 11 (1.4%), ergots in 13 (1.6%), others in 10 (1.3%), and unknown in 21 (2.6%). Among patients overusing multiple medications (*n* = 439), 253 (57.6%), 132 (30.1%), and 54 (12.3%) used two, three, and four or more medications, respectively, and the mostly commonly used combinations include acetaminophen plus combination analgesics (*n* = 104, 23.7%), acetaminophen plus NSAIDs (*n* = 39, 8.9%), acetaminophen plus ergots (*n* = 21, 4.8%), etc.

**Table 1:** Clinical characteristics in chronic migraine patients with and without medication-overuse headache

	CM (*n* = 1419)	Without MOH (*n* = 620)	With MOH (*n* = 799)	*p* value
Age	41.7 ± 13.9	40.6 ± 14.6	42.5 ± 13.2	0.008
Female gender	80.0%	79.8%	80.1%	0.903
Age at migraine onset (years)	21.8 ± 10.7	23.1 ± 11.3	20.8 ± 10.0	< 0.001
Duration of CM (months)	78.6 ± 107.1	59.7 ± 99.5	92.9 ± 110.4	< 0.001
Age at CM onset (years)	35.6 ± 14.0	36.0 ± 14.7	35.3 ± 13.4	0.370
Time from migraine onset to CM onset (years)	14.1 ± 11.7	13.1 ± 11.8	14.9 ± 11.5	0.007
Average headache severity (/10)	6.7 ± 2.0	6.1 ± 1.9	7.1 ± 1.9	< 0.001
Monthly headache days	23.3 ± 6.6	23.0 ± 6.7	23.6 ± 6.5	0.069
Monthly analgesic use (days/month)	12.8 ± 10.4	4.0 ± 5.5	19.7 ± 7.8	< 0.001
Hypnotic use ≥ 3 days/week	32.0%	21.0%	40.5%	< 0.001
Tobacco use	13.6%	9.1%	17.2%	< 0.001
Alcohol use	32.0%	33.7%	30.6%	0.220
Caffeine use	65.1%	64.2%	65.1%	0.519
HIT-6	63.8 ± 7.7	62.5 ± 7.0	64.8 ± 8.1	< 0.001
HADS-A	9.4 ± 4.5	9.3 ± 4.5	9.5 ± 4.6	0.385
HADS-D	7.8 ± 4.5	7.4 ± 4.4	8.1 ± 4.6	0.002
PSQI	11.5 ± 4.3	11.0 ± 4.1	12.0 ± 4.3	< 0.001
LDQ	8.9 ± 8.1	3.9 ± 5.0	12.8 ± 7.8	< 0.001
SDS	4.9 ± 4.1	2.3 ± 2.8	6.9 ± 3.9	< 0.001

*Abbreviations*: *CM* Chronic migraine, *HADS* Hospital Anxiety and Depression Scale (A = anxiety subscale, D = depression subscale), *HIT-6* Headache Impact Test-6, *LDQ* Leeds Dependence Questionnaire, *MOH* Medication-overuse headache, *PSQI* Pittsburgh Sleep Quality Index, *SDS* Severity of Dependence Scale

Patient with MOH were older than those without (mean age: 42.5 ± 13.2 vs. 40.6 ± 14.6 years, *p* = 0.008) (Table 1), and had an earlier onset of migraine (onset age 20.8 ± 10.0 vs. 23.1 ± 11.3 years, *p* < 0.001), a longer duration of CM (92.9 ± 110.4 vs. 59.7 ± 99.5 months, *p* < 0.001), greater average headache intensity (7.1 ± 1.9 vs. 6.1 ± 1.9, *p* < 0.001), a higher frequency of acute medication use (19.7 ± 7.8 vs. 4.0 ± 5.5 days/month, *p* < 0.001), a greater headache impact (HIT-6: 64.8 ± 8.1 vs. 62.5 ± 7.0, *p* < 0.001), more symptoms of depression (HADS-D: 8.1 ± 4.6 vs. 7.4 ± 4.4, *p* = 0.002), and poorer sleep quality (PSQI: 12.0 ± 4.3 vs. 11.0 ± 4.1, *p* < 0.001). Besides, patients with MOH had higher total scores on the LDQ (12.8 ± 7.8 vs. 3.9 ± 5.0, *p* < 0.001) and the SDS (6.9 ± 3.9 vs. 2.3 ± 2.8, *p* < 0.001) than those without (Table 1).

### Association between smoking and MOH, especially in men

Tobacco use was more common in patients with MOH (17.2% vs. 9.1%, *p* < 0.001) than in those without, but the proportions of patients reporting current use of alcohol (30.6% vs. 33.7%, *p* = 0.220) or caffeinated beverages (65.1% vs. 64.2%, *p* = 0.519) were similar. In univariate analysis, smoking was associated with increased odds of MOH (OR = 2.08 [95% CI = 1.49–2.89], *p* < 0.001). There was a significant sex-by-smoking interaction (OR = 2.33 [1.08–5.03], *p* = 0.031), and the association was stronger in men (OR = 3.87 [1.99–7.54], *p* < 0.001) than in women (OR = 1.66 [1.13–2.44], *p* = 0.010). After taking age, the number of monthly headache days, CM duration (years), and scores on HADS-A, HADS-D, and PSQI into consideration, there was still a significantly sex-by-smoking interaction (OR = 2.63 [1.16–5.98], *p* = 0.021), and the association between smoking and MOH remained significant in men (OR = 3.60 [1.73–7.50], *p* = 0.001), but not in women (OR = 1.34 [0.88–2.04], *p* = 0.171) (Table 2). The findings were similar when either LDQ or SDS scores were further included in the model (data not shown). However, current use of alcohol or caffeinated beverages was not associated with the presence of MOH (data not shown).

**Table 2:** Risks of MOH in relation to current use of tobacco

	Total		Sex-by-smoking interaction		Men		Women	
	OR (95% CI)	*p* value	OR (95% CI)	*p* value	OR (95% CI)	*p* value	OR (95% CI)	*p* value
Tobacco use	1.79 (1.25–2.56)	0.002	1.35 (0.89–2.06)	0.157	3.60 (1.73–7.50)	0.001	1.34 (0.88–2.04)	0.171
Male sex	1.06 (0.79–1.41)	0.712	0.91 (0.67–1.25)	0.563				
Age (years)	1.00 (0.99–1.01)	0.528	1.00 (0.99–1.01)	0.602	1.00 (0.98–1.02)	0.968	1.00 (0.99–1.01)	0.622
Monthly headache days	1.00 (0.98–1.02)	0.873	1.00 (0.98–1.02)	0.977	0.97 (0.94–1.02)	0.207	1.01 (0.99–1.03)	0.494
CM duration (years)	1.03 (1.01–1.05)	< 0.001	1.03 (1.01–1.05)	< 0.001	1.13 (1.06–1.19)	< 0.001	1.02 (1.00–1.03)	0.033
Average headache intensity	1.24 (1.16–1.32)	< 0.001	1.24 (1.16–1.32)	< 0.001	1.17 (1.01–1.36)	0.038	1.26 (1.18–1.35)	< 0.001
HADS-A	0.98 (0.94–1.01)	0.154	0.98 (0.94–1.01)	0.152	0.94 (0.76–1.02)	0.129	0.99 (0.95–1.03)	0.498
HADS-D	1.03 (1.00–1.07)	0.091	1.03 (1.00–1.07)	0.090	1.05 (0.97–1.15)	0.232	1.03 (0.99–1.07)	0.198
PSQI	1.03 (1.00–1.06)	0.087	1.03 (1.00–1.06)	0.070	1.03 (0.96–1.11)	0.461	1.03 (0.99–1.06)	0.120
Sex-by-smoking interaction			2.63 (1.16–5.98)	0.021				

*Abbreviations*: *CM* Chronic migraine, *HADS* Hospital Anxiety and Depression Scale (A = anxiety subscale, D = depression subscale), *MOH* medication-overuse headache, *PSQI* Pittsburgh Sleep Quality Index

On the other hand, frequent hypnotic use, i.e. ≥ 3 days/week, was more common in patients with MOH (40.5% vs. 21.0%, *p* < 0.001) than in those without, and was associated with increased odds of having MOH (OR = 2.55 [95% confidence interval = 2.00–3.24], *p* < 0.001). However, there was no significant sex difference (OR = 1.31 [0.68–2.52], *p* = 0.426 for sex-by-frequent-hypnotic-use interaction). The findings were similar after controlling for potential confounders (data not shown).

### Diagnostic utilities of LDQ and SDS for MOH in women and men

**Fig. 2:**
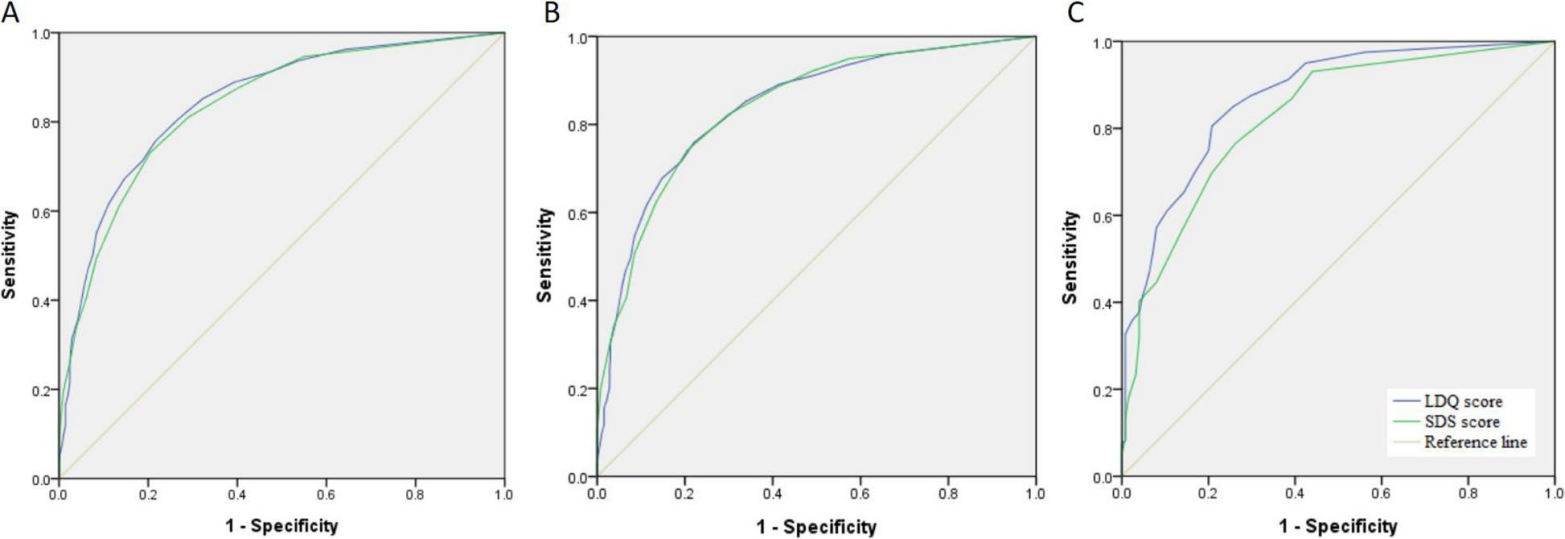
Receiver Operating Characteristic (ROC) curves of the LDQ and the SDS scores predictive of MOH in the entire study population **A**, in women **B**, and in men **C**

In the entire study population, the cutoff score of the LDQ for a diagnosis of MOH was determined at 7, with a sensitivity of 75.7% and a specificity of 78.4% (AUC = 0.85), and the cutoff score of the SDS was determined at 5 (sensitivity = 73.1%, specificity = 79.5%, AUC = 0.84) (Fig. 2A).

When women and men were analyzed separately, the cutoff scores of the LDQ for MOH were determined at 7 for women (sensitivity = 75.9%, specificity = 78.0%, AUC = 0.84) and 6 for men (sensitivity = 80.5%, specificity = 79.2%, AUC = 0.87) (Fig. 2B-C), and the cutoff scores for the SDS were 5 for women (sensitivity = 74.0%, specificity = 79.6%, AUC = 0.84) and 4 for men (sensitivity = 76.7%, specificity = 73.6%, AUC = 0.83).

### Acute medication use and clinical correlations in patients with MOH

When individual acute medications were analyzed separately, the most commonly used acute medications were acetaminophen (65.8%), NSAIDs (58.2%), ergots (19.6%), and triptans (13.1%). NSAID use was more frequent in women than in men (60.3% vs. 49.7%, *p* = 0.015), whereas men were more likely to use cold syrups (13.2% vs. 7.8%, *p* = 0.032) and tramadol (11.3% vs. 6.1%, *p* = 0.022) (Table 3). Triptan users scored higher on the LDQ (14.8 ± 7.7 vs. 12.5 ± 7.8, *p* = 0.004) and the SDS (7.9 ± 3.8 vs. 6.8 ± 3.9, *p* = 0.008) when compared with non-users. The findings were similar for ergot users (LDQ: 14.5 ± 7.8 vs. 12.4 ± 7.7, *p* = 0.003; SDS: 8.1 ± 4.0 vs. 6.7 ± 3.8, *p* < 0.001). On the other hand, acetaminophen use was associated with lower SDS scores (6.7 ± 3.9 vs. 7.4 ± 3.9, *p* = 0.021), although the LDQ scores were comparable (12.8 ± 7.8 vs. 12.9 ± 7.8, *p* = 0.777). There was no difference for other acute medication, and the trends were generally consistent between the sexes (data not shown).

**Table 3:** Sex differences in clinical presentations of MOH

	Female (*n* = 640)	Male (*n* = 159)	*p* value
Age	43.0 ± 13.1	40.5 ± 13.4	0.030
Age at migraine onset (years)	20.7 ± 10.0	21.0 ± 10.2	0.685
Duration of CM (months)	93.2 ± 112.4	91.5 ± 103.0	0.432
Age at CM onset (years)	35.8 ± 13.8	33.3 ± 12.7	0.041
Time from migraine onset to CM onset (years)	15.4 ± 11.5	12.9 ± 11.1	0.008
Average headache severity (/10)	7.2 ± 1.9	6.7 ± 1.9	0.005
Monthly headache days	23.7 ± 6.7	23.2 ± 6.7	0.318
Monthly analgesic use (days/month)	19.7 ± 7.8	19.6 ± 7.7	0.962
Acute medication overused (non-exclusive)^a^			
Acetaminophen	65.9%	65.4%	0.900
NSAIDs	60.3%	49.7%	0.015
Ergots	19.5%	20.1%	0.866
Triptans	13.0%	13.8%	0.772
Cold syrups	7.8%	13.2%	0.032
Tramadol	6.1%	11.3%	0.022
Hypnotic use ≥ 3 days/week	42.2%	33.5%	0.048
Tobacco use	13.7%	31.0%	< 0.001
Alcohol use	27.1%	44.7%	< 0.001
Caffeine use	65.7%	66.2%	0.902
Headache characteristics			
Pulsatile	72.8%	68.6%	0.285
Unilateral	72.3%	74.8%	0.526
Aggravation by physical activities	88.8%	84.3%	0.115
Nausea	89.5%	81.1%	0.004
Vomiting	49.4%	37.7%	0.008
Photophobia	55.2%	48.4%	0.128
Phonophobia	83.9%	77.4%	0.051
HIT-6	65.1 ± 8.0	63.4 ± 8.3	0.009
HADS-A	9.7 ± 4.5	8.9 ± 4.7	0.062
HADS-D	8.0 ± 4.7	8.4 ± 4.5	0.391
PSQI	12.2 ± 4.3	10.9 ± 4.4	0.001
LDQ	12.7 ± 7.7	13.2 ± 8.2	0.460
SDS	7.0 ± 3.9	6.5 ± 4.0	0.503

*Abbreviations*: *CM* Chronic migraine, *HADS* Hospital Anxiety and Depression Scale (A = anxiety subscale, D = depression subscale), *HIT-6* Headache Impact Test-6, *LDQ* Leeds Dependence Questionnaire, *PSQI* Pittsburgh Sleep Quality Index, *SDS* Severity of Dependence Scale

^a^Percentages were expressed as proportions of patients using a particular category of medication alone or in combination with other acute medication(s)

### Sex differences in clinical manifestations of patients with MOH

When compared with men, women with MOH were older (mean age: 43.0 ± 13.1 vs. 40.5 ± 13.4 years, *p* = 0.030), had greater average headache intensity (7.2 ± 1.9 vs. 6.7 ± 1.9, *p* = 0.005) and headache impact (HIT-6: 65.1 ± 8.0 vs. 63.4 ± 8.3, *p* = 0.009), and poorer sleep (PSQI: 12.2 ± 4.3 vs. 10.9 ± 4.4, *p* = 0.001), and were more likely to have frequent hypnotic use (i. e. ≥ 3 days/week) (42.2% vs. 33.5%, *p* = 0.048) (Table 3). Besides, women were more likely to have nausea (89.5% vs. 81.1%, *p* = 0.004) and vomiting (49.4% vs. 37.7%, *p* = 0.008) as headache-associated symptoms. On the other hand, the latency between migraine onset and CM onset was shorter in men than in women (12.9 ± 11.1 vs. 15.4 ± 11.5 years, *p* = 0.008). The proportions of patients reporting tobacco (31.0% vs. 13.7%, *p* < 0.001) or alcohol (44.7% vs. 27.1%, *p* < 0.001) use were higher in men than in women with MOH. However, other clinical headache features and associated symptoms were similar between the sexes.

In exploratory analysis based on sex, female MOH patients with current tobacco use had more frequent acute medication use (22.0 ± 7.3 vs. 19.3 ± 7.8 days/month, *p* = 0.002) and shorter intervals between migraine onset and CM onset (12.7 ± 9.4 vs. 15.6 ± 11.7 years, *p* = 0.019) than those without, despite similar age at migraine onset (20.3 ± 9.3 vs. 20.7 ± 10.1 years, *p* = 0.713) and headache frequencies (24.5 ± 6.2 vs. 23.6 ± 6.4 days/month, *p* = 0.235). On the other hand, the frequencies of acute medication use (19.0 ± 7.9 vs. 20.0 ± 7.6 days/month, *p* = 0.433) and intervals between migraine onset and CM onset (12.7 ± 10.6 vs. 12.8 ± 11.4 years, *p* = 0.964) were comparable between men with MOH who smoked and who did not, and so were the ages at migraine onset and headache frequencies (data not shown).

## Discussion

In the current study, it was found that smoking was independently associated with MOH in men, but not in women, and frequent hypnotic use was associated with MOH, regardless of sex. Besides, the LDQ and the SDS were of excellent diagnostic performances for the diagnosis of MOH in patients with CM of both sexes, although the cutoff scores for both of the instruments were one point lower in men than in women. In addition, MOH in the current cohort was characterized by a more severe phenotype in women, and a shorter latency between migraine onset and CM onset in men. Sex should be considered as an important factor in the initial evaluation of patients with suspected MOH.

Important strengths of the present study included sample size, quality of the data, and study design. The current study recruited more than 1400 consecutive CM patients. Therefore, selection bias could be reduced, and more accurate estimates could be derived. In particular, the number of male patients in the present study was larger than those in the majority of prior studies. Besides, the data were of high quality and reliability. The diagnoses of CM and MOH were made by headache specialists according to the ICHD-3 criteria. The data were collected systematically by using a specifically designed questionnaire. In addition, the clinical utilities of the LDQ and the SDS in CM patients with and without MOH were compared directly. In addition, dependence behaviors and related manifestations were compared between the sexes. Therefore, the present study could provide more comprehensive and complete information that is more relevant to CM patients evaluated in the clinical settings.

It was found that smoking was associated with MOH, and the association was stronger in men. An association between smoking and MOH, as well as SUDs, was reported in the literature [19, 35–37], although whether there could be sex differences has rarely been discussed upon. Since smoking shares some clinical features with SUDs, it is possible that tobacco use could reflect the presence of disturbances in the reward circuit such that these patients were at risk of developing MOH. On the other hand, frequent hypnotic use was also associated with MOH, which was in keeping with the association between MOH and tranquilizers reported in the literature [19, 38]. However, there was no sex difference. Interpretations for the associations with alcohol and caffeine use could be more complicated since these agents could have direct impacts on migraine attacks [39]. In addition to the mechanisms related to reward circuit, it is also possible that the identified association between MOH and smoking and hypnotic use could also be attributed to certain shared factors, such as socioeconomic status, stress levels, lifestyles, etc. Further studies are needed to clarify the roles of these potential confounders. Interestingly, smoking cessation could have a positive effect on the treatment outcome of SUDs [40]. Whether smoking cessation would reduce the risk or improve the prognosis of MOH, especially in men, deserves further exploration.

The current MOH cohort was characterized by a low rate of triptan use, and there was an association between overuse of triptans or ergots and more severe dependence behaviors. The rate of triptan use or overuse in our MOH patients was lower than estimates from some studies [41–43], although the trend was consistent with prior reports from Taiwan, which could be attributed to the costs of tritpans and the restrictions on their use, e.g. ≤ 400 mg/month for sumatriptan tablets, by the reimbursement regulations of the Taiwan NHI [29, 44]. In the present study, triptan or ergot users scored higher on the LDQ and the SDS. In fact, it takes shorter latencies and lower doses for patients overusing triptans or ergots to develop MOH than those with simple analgesic overuse [41]. These findings could be suggestive of an association between overuse of these two classes of acute medications and a greater tendency to develop MOH. Alternatively, it is also possible that patients with different headache severities or personality traits would preferably overuse certain categories of acute medications [45, 46]. However, interpretations could be cautious since the categories of triptan or ergot overuse included patients using these agents alone or in combination with other acute medications. More studies are needed to provide more insight into the underlying pathophysiology.

In the current study, the LDQ and the SDS were of comparable accuracies in the detection of MOH in CM patients of both sexes. It was reported that the severity of dependence behaviors, as measured by the LDQ, in CDH patients with MO was greater than those with episodic primary headache disorders, and was similar to that in SUDs involving alcohol or illicit drugs [23]. Besides, it was demonstrated that an SDS score of ≥ 5 was correlated with the presence of MO in the general population [25], as well as behaviors of substance dependence in the clinical settings [17], and the cutoff score identified in our study was the same. In MOH patients who received detoxification, responders had greater decrease in the LDQ scores [27]. Besides, the SDS scores at baseline were predictive of the outcome in MOH associated with primary headache disorders [28], and the SDS could decrease following withdrawal of acute medications [47]. In addition to preventive medications, behavioral therapy targeting at dependence behaviors could also be an important treatment option to improve the treatment outcome [14]. It was demonstrated that brief intervention, which consisted of feedback based on the initial scores of SDS and consultations to reduce the use of acute medication, was an effective for MOH patients [48]. It is possible that both the LDQ and the SDS could have important roles not only as screening instruments, but also in the treatment and prognostication.

In the present study, it was found that cutoff scores for the diagnosis of MOH were lower in men than in women for both of the instruments, and there were differences in clinical manifestations of MOH and medication use between the sexes. Actually, it was demonstrated in a population-based study that SDS ≥ 5 for women and ≥ 4 for men could help detect MO in a group of patients with primary chronic headache disorders, of whom 95% had chronic TTH [25], and the cutoffs identified in our study were exactly the same. In fact, there have been some reports on sex-specific cutoffs in the diagnosis a variety of diseases, such as right bundle branch block, myocardial infarction, venous thromboembolism, etc. [49–52], which indicate that an uniform diagnostic cutoff score may not always be the best strategy. On the other hand, it was found that MOH in women was associated with more severe clinical manifestations, which is consistent with the trend in migraine and CM [9, 12, 53]. Besides, MOH in men was characterized by a shorter interval between migraine onset and CM onset. In fact, among patients with EM at baseline, men were more likely to have CM onset at 6, 9, and 12 months in the CaMEO study [54]. This might indicate that migraine chronification could take a shorter period of time to develop in men. Since there is a female preponderance in both CM and MOH [11–13], data in male patients could be under-presented in the literature. Clinical decisions for men with MOH based on available evidence could be biased. Further studies are needed to provide more insights on sex-specific differences on such an issue.

There are some limitations. First of all, the patients were recruited from the headache clinic of a tertiary medical center, and only patients who were 20–65 years old were included in the study. There could be concerns about generalizability. However, there is no strict regulation on the process of referral in the healthcare system of our country, and the majority of patients came in directly without referral. Therefore, patients involved in the present study could reflect those in the general population to a certain extent. Second, both the LDQ and the SDS were administered by the patients themselves, and there could be concerns about reliability. However, the responses in these instruments were verified by headache specialists at face-to-face interviews. Besides, the self-administered version of SDS has been validated against headache specialist interviews by telephone [55]. Third, dependence behaviors in the current study were defined based on the scores on neuropsychological instruments, rather than formal psychiatric evaluation. In fact, although MOH shares many clinical and neuroimaging features with SUDs, certain important elements of SUD are not seen in patients with MOH, such as craving, drug-associated cues, impulsivity, and compulsive behaviors [56, 57]. Besides, controversies remain as to whether overuse of acute medications could just reflect the severity of the underlying headache disorders [45], since most of the commonly used acute medications are generally considered to be of low abuse potential [58, 59]. Therefore, the findings should be interpreted with caution. Fourth, the use of tobacco, alcohol, and caffeinated beverages was defined by dichotomous variables, and whether the amount or frequency of their use to have an impact is yet to be determined. Finally, as a cross-sectional study, only associations, rather than causal relationships, could be identified, and the findings should be interpreted with caution.

In conclusion, the present study demonstrated differential risks of MOH associated with tobacco use between men and women and identified sex-specific cutoff scores of the LDQ and the SDS for MOH among patients with CM. Besides, compared with men, women with MOH seemed to have longer latencies of migraine chronification, except for those who smoke. Sex should be taken into account in the evaluation of MOH.

## Data Availability

The datasets generated and/or analyzed during the current study are not publicly available due to participant confidentiality.

## Abbreviations

AUC: Area under curve
CI: Confidence interval
CaMEO: Chronic Migraine Epidemiology and Outcomes
CDH: Chronic daily headache
CM: Chronic migraine
HADS: Hospital Anxiety and Depression Scale
HADS-A: Anxiety subscale of Hospital Anxiety and Depression Scale
HADS-D: Depression subscale of Hospital Anxiety and Depression Scale
HIT-6: Headache Impact Test-6
ICHD-3: Third Edition of the International Classification of Headache Disorders
LDQ: Leeds Dependence Questionnaire
MOH: Medication-overuse headache
OR: Odds ratio
ROC: Receiver operating curve
SDS: Severity of Dependence Scale
SUD: Substance use disorder
TTH: Tension-type headache
